# Modest additive effects of integrated vector control measures on malaria prevalence and transmission in western Kenya

**DOI:** 10.1186/1475-2875-12-256

**Published:** 2013-07-19

**Authors:** Guofa Zhou, Yaw A Afrane, Amruta Dixit, Harrysone E Atieli, Ming-Chieh Lee, Christine L Wanjala, Leila B Beilhe, Andrew K Githeko, Guiyun Yan

**Affiliations:** 1 Program in Public Health, University of California, Irvine, USA; 2 Centre for Global Health Research, Kenya Medical Research Institute, Kisumu, Kenya; 3 CIRAD Bios – UR Bioagresseurs, Analyse et Gestion du Risque (106), Montpellier, Cameroon

**Keywords:** Insecticide-treated bed-nets, Indoor residual spraying, Larvicide, Integration, Parasite prevalence, Malaria incidence rate, Vector density, Evaluation, Effectiveness

## Abstract

**Background:**

The effect of integrating vector larval intervention on malaria transmission is unknown when insecticide-treated bed-net (ITN) coverage is very high, and the optimal indicator for intervention evaluation needs to be determined when transmission is low.

**Methods:**

A post hoc assignment of intervention-control cluster design was used to assess the added effect of both indoor residual spraying (IRS) and *Bacillus*-based larvicides (Bti) in addition to ITN in the western Kenyan highlands in 2010 and 2011. Cross-sectional, mass parasite screenings, adult vector populations, and cohort of active case surveillance (ACS) were conducted before and after the intervention in three study sites with two- to three-paired intervention-control clusters at each site each year. The effect of larviciding, IRS, ITNs and other determinants of malaria risk was assessed by means of mixed estimating methods.

**Results:**

Average ITN coverage increased from 41% in 2010 to 92% in 2011 in the study sites. IRS intervention had significant added impact on reducing vector density in 2010 but the impact was modest in 2011. The effect of IRS on reducing parasite prevalence was significant in 2011 but was seasonal specific in 2010. ITN was significantly associated with parasite densities in 2010 but IRS application was significantly correlated with reduced gametocyte density in 2011. IRS application reduced about half of the clinical malaria cases in 2010 and about one-third in 2011 compare to non-intervention areas.

**Conclusion:**

Compared with a similar study conducted in 2005, the efficacy of the current integrated vector control with ITN, IRS, and Bti reduced three- to five-fold despite high ITN coverage, reflecting a modest added impact on malaria transmission. Additional strategies need to be developed to further reduce malaria transmission.

## Background

Malaria is one of the most devastating tropical infectious diseases in the world. In 2010, there were 216 million episodes of malaria worldwide and 655,000 malaria deaths, with the vast majority of the episodes and deaths in Africa, and 86% of malaria deaths being children under five years of age [1]. Significant progress has been made in fighting malaria during the past decade. Malaria cases and deaths have declined as a result of intensified intervention including the scaled up use of insecticide-treated bed nets including long-lasting insecticidal nets (ITNs), artemisinin-combination therapy (ACT), and indoor residual spraying (IRS) , as well as other intervention measures [1,2]. ITNs are known to be highly effective in reducing malaria transmission and the scaling-up of ITNs has experienced two major milestones in the past decade [3]. ITNs have been used in rural Africa since the early 1980s, but the coverage had been low [4]. The percentage of households owning at least one ITN in sub-Saharan Africa was estimated to be 3% in 2000 [1]. In early 2000, ITNs were delivered through the commercial retail sector, mainly targeting pregnant women and children under five years of age. However, net coverage was persistently low by mid-2000 [5]. In 2006, the Global Fund to fight HIV/AIDS, Tuberculosis and Malaria, along with other donors, supported a massive community-based distribution of ITNs in Africa, resulting in the mass distribution of nets provided either free of charge (for pregnant women and children under five) or heavily subsidized through health facilities [6,7]. In light of the downward trends in reported malaria cases, the Roll Back Malaria Partnership had set the year 2010 as the date to achieve universal coverage for all populations at risk of malaria, leading to the 2011 mass ITN distribution campaign [1]. This campaign delivered ITNs free of charge to everybody at risk for malaria. The percentage of households owning at least one ITN in sub-Saharan Africa was estimated to be 50% before the 2011 mass ITN distribution campaign [1]. The current ITN coverage and its impact on malaria transmission are subject to further investigation.

IRS has also been proven to be an alternative intervention measure that could significantly reduce malaria transmission [8]. The past trials (when ITN coverage was very low) of mono-IRS application have resulted in similar reductions in vector densities and parasite prevalence to mono-ITN implementations, but the impact of IRS on reducing malaria transmission is short-lived compared to ITNs [8,9]. Interest in IRS has been rekindled in recent years, as it is increasingly considered to be a key component of integrated malaria management. Regular spraying of each human dwelling becomes less practical as the size of the control area increases. Around malaria transmission focal points however, targeted IRS may pose a feasible alternative to mass spraying. Several experimental trials have proven that targeted IRS is feasible and effective in reducing malaria transmission [7,10,11]. Recently, larval control techniques such as breeding habitat management and larvicide application have been implemented in several trial experiments. Larval control has been suggested as one of the major components of integrated malaria management [12,13]. All previous studies used larval density as an indicator to measure the reduction in vector population, but larval density does not necessarily correspond with the same level of indoor resting density because larval survivorship is usually very low in natural conditions and varies tremendously among different habitat types [14].

More importantly, previous tests of IRS and larval control were all conducted when ITN coverage was relatively low and vector densities were high. As ITN coverage increases, vector density, especially indoor resting density, will decrease. Modelling simulation results suggested that local malaria transmission could be interrupted by ITN alone if ITN coverage is >80% [15,16]. Analysis of long-term clinical records showed that the resilience of malaria transmission patterns to environmental variation could be mediated even if ITN coverage was as low as 20% [17]. The question lies in the degree of impact that IRS and larval control have on reducing vector density and parasite infection in addition to ITN when ITN coverage is moderate (~50%) or very high (>80%). Furthermore, to achieve sustainable impact, regular formulation larvicide must be applied weekly and IRS must be repeated every six months [13,18]. These limitations pose the challenges of cost and feasibility for implementing IRS and larvicide intervention on a large scale. Alternatively, the hotspot transmission area can be targeted before the transmission peak season so as to delay or eliminate the build up of vector population, which may, in turn, significantly reduce parasite transmission. In spite of the promising results in reducing malaria transmission from the trial implementation of targeted IRS when ITN coverage was very low, studies addressing the impact of IRS intervention on malaria transmission, in situations where ITN coverage is high, have remained rudimentary until recently [7,10].

In order to investigate the impact of ITN coverage on the effect of IRS and larval control, both low and high coverage of ITNs are required. In this context, the trial interventions conducted in 2010 and 2011 provided a unique and robust opportunity. This report analysed how ITN coverage mediated the effect of IRS and, more importantly, what indicators are more sensitive for the evaluation of the intervention when transmission intensity becomes low.

## Methods

### Study site and spatial randomization

**Figure 1:**
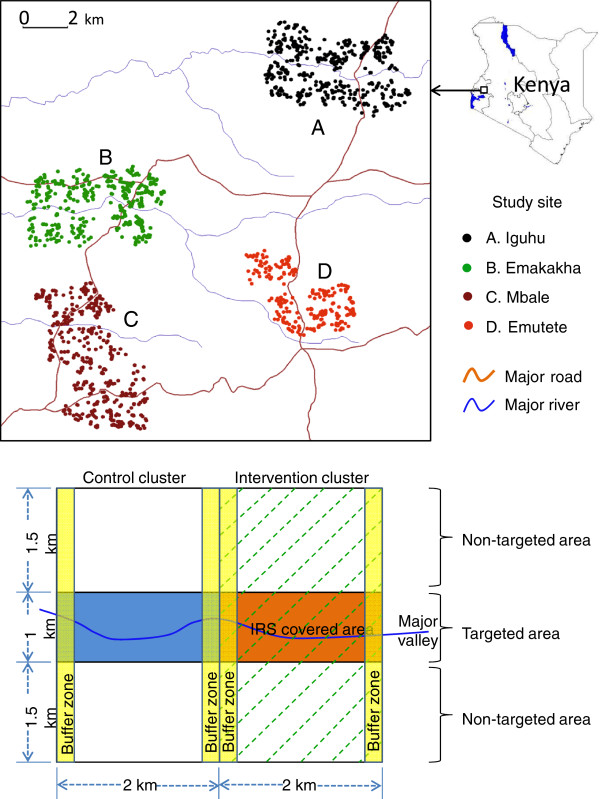
**Top panel: the study sites; Bottom panel: study design.** Illustration of a typical pair of intervention (right) and control (left) clusters with randomized pair-wise experimental design. Bti application (green dash-lined) covered the entire intervention cluster. Each cluster was divided into targeted (coloured area) and non-targeted (no background colour) areas and IRS was only applied in the targeted area within the intervention clusters. Buffer zones (250 m in width) are the areas where samples were not included in final analysis.

The study was conducted in four regions in the highlands of western Kenya: Iguhu (34˚44΄ E, 0˚11΄ N, 1,430-1,580 m above sea level) in Kakamega county (previously Kakamega district); Mbale (34˚43΄ E, 0˚05΄ N, 1,520-1,640 m above sea level) in Vihiga county; Emutete (34˚38΄ E, 0˚02΄ N, 1,480-1,640 m above sea level); and Emakakha (34˚39΄ E, 0˚07΄ N, 1,460-1,520 m above sea level) in Emuhaya county (Figure 1, top panel). The 2010 intervention trial was conducted in Iguhu, Emutete and Mbale; due to logistic reasons Emakakha replaced Mbale as a 2011 intervention site. The climatic, topographic, and ecological characteristics of Iguhu, Mbale, and Emutete have been described in previous studies [5,19,20]. The topographic and ecological characteristics in Emakakha are comparable to that in Emutete. Briefly, December to February is typically the low malaria transmission season and May to July is the peak transmission season in western Kenya. The differences in vector densities and parasite prevalence among Iguhu, Emutete, and Mbale sites have been studied previously; however, the condition of malaria transmission in the Emakakha area is largely unknown [5,19,20].

All houses in the study sites were identified using 1 m resolution Ikonos images. Each house was assigned a unique identification number. Participating households were randomly selected from these identified houses. All houses were mapped on colour Ikonos image, which are used by field surveys.

### Mixed intervention-control design

Malaria intervention methods include targeted IRS of lambda-cyhalothrin (ICON™) in 2010 and both IRS and larval control using larvicide *Bacillus thuringiensis israelensis* (Bti) in 2011. Nine paired clusters were selected from three study sites in 2010. Each site had three paired clusters, one cluster of each pair was randomly selected for intervention and the other was used as control. Each cluster was divided into targeted and non-targeted sections (Figure 1, bottom panel). The size of each cluster is approximately 2 sq km. Clusters were paired up based on their similarity in ecological settings and entomological and parasitological survey results prior to intervention. The selection of targeted IRS area within each cluster was not randomized; rather it was based on the results of past studies and baseline surveys prior to the intervention, i.e., targeted on major malaria transmission area – highest vector density and parasite prevalence [7]. The design was similar to a previous study, with the targeted IRS area being approximately one quarter of the intervention cluster [7]. The 2011 intervention trial design was a modified version of the one implemented in 2010, with only six pairs of study clusters, each measuring 2 × 3 km. The criteria of selecting paired clusters and intervention areas were the same as in 2010. The major change in the 2011 trial was the application of Bti in the entire intervention cluster in addition to targeted IRS.

### IRS intervention

Lambda-cyhalothrin (brand name ICON), a synthetic pyrethroid insecticide which is among the insecticides recommended by the World Health Organization and National Malaria Control Board of Kenya [7], was applied only in the targeted section of the intervention clusters in March 2010 and March 2011, prior to the long rainy season that triggers an increase in the density of malaria vectors and signals the beginning of the peak malaria transmission period in this highland area. All houses in the intervention area were identified, marked, and numbered using satellite images, and were sprayed with lambda-cyhalothrin. The interior walls and roofs of the targeted houses were sprayed. Numbers of houses sprayed at each site in both 2010 and 2011 are shown in Table 1. No other IRS techniques were utilized in the study areas during the two-year study period.

**Table 1:** Study sites and number of households/individuals participated in the study by site and by study time in 2010 and 2011

**Type and time of survey**^**†**^	**Iguhu**	**Emutete**	**Mbale/ Emakakha**^**‡**^	**Total (clusters)**^**§**^
ICON 2010 March (HH)	616	1,082	1,388	3,086 (18)
ICON 2011 March (HH)	529	1,169	1,148	2,846 (12)
PSC 2010 Feb (HH)	382	355	340	1,077 (18)
PSC 2010 May (HH)	332	354	332	1,018 (18)
PSC 2010 Jul (HH)	358	424	394	1,176 (18)
PSC 2011 Feb (HH)	417	343	400	1,160 (12)
PSC 2011 May (HH)	506	460	449	1,415 (12)
PSC 2011 Jul (HH)	484	429	429	1,342 (12)
APS 2010 Feb (Ind)	798	1,157	951	2,906 (18)
APS 2010 May (Ind)	728	1,014	1,140	2,884 (18)
APS 2011 Feb (Ind)	1,893	1,838	1,408	5,139 (12)
APS 2011 May (Ind)	1,494	1,587	1,602	4,683 (12)
APS 2011 Jul (Ind)	1,360	1,356	1,587	4,323 (12)
ACS 2010 Cohort (Ind)	1,760	1,794	2,674	6,248 (18)
ACS 2011 Cohort (Ind)	2,019	1,697	1,858	5,574 (12)
ITN 2010 ownership (HH)	321	354	322	997
ITN 2010 coverage (Ind)	991	1,442	1,296	3,702
ITN 2011 ownership (HH)	483	460	447	1,390
ITN 2011 coverage (Ind)	1,745	2,175	1,767	5,687

† HH = number of households; Ind = number of individuals.

‡ Mbale is a 2010 study site and Emakakha is a 2011 study site as a replacement of Mbale.

§ Clusters were equally divided among three sites and ITN surveys were based on study site.

### Larvicide application

The working hypothesis is that application of Bti before the onset of long rainy season, when used in partnership with IRS/ITN interventions, can substantially suppress early season larval population, thereby reducing subsequent vector population build-up during the long rainy season. For that reason, the first round application of Bti was completed in February/March, 2011, and the second in March/April, 2011, about four weeks after the first application. Aquatic habitats were searched thoroughly by a team of technicians accompanied by field assistants from local villages. Bti granules (CG formulation, VectoMax, Valent BioSciences Corp, Illinois, USA) were applied, following guidelines provided by manufacturer, in all aquatic habitats in all intervention clusters, regardless of habitat type, size, and presence/absence of mosquito larvae. No other Bti or other larval control interventions were implemented in all study sites in 2010 and 2011.

### Intervention evaluation

#### ITN coverage

Field surveys showed that household ITN ownership in the Iguhu study site was 13% and bed net coverage was 5% of the surveyed population (assuming that one bed net covers two individuals) in 2004 [7]. The Global Fund-supported, community-wide mass distribution of ITNs was completed in June-September 2006 in all study sites, and the second round of mass ITN distribution was accomplished during May-July 2011. The 2006 mass ITN distribution targeted children under five years of age and pregnant women. The 2011 mass ITN campaign distributed ITNs to all persons at risk of malaria free of charge, thus this campaign has very high potential to increase the ITN coverage in the population. Cross-sectional household level bed net ownership and individual level bed net usage surveys were conducted in May 2010 and June 2011 in all study sites. At each study site, between 350–400 and 450–500 houses were randomly selected from survey maps in 2010 and 2011, respectively. Due to mismatches between satellite image and actual house location and/or logistic reasons, the actual number of houses surveyed varied. The average sample size was 332 households (ranging from 321–354) per site in 2010 and 463 (ranging from 447–483) households per site in 2011. The average size of the surveyed population was 1,234 individuals (ranging from 991–1,442) per site in 2010 and 1,896 individuals (ranging from 1,745–2,175) per site in 2011. Information on bed net ownership, number of ITNs owned, individuals who used bed nets, and demographics were included in the recording forms.

Household ITN ownership rate is defined as proportion of households owning at least one ITN regardless of the type and condition of the nets. Population ITN coverage rate is defined as the proportion of individuals, in the population surveyed, who reported to own ITN regardless of usage. ITN usage is defined as the proportion of individuals who reported to be sleeping under bed nets the night before the survey. Actual ITN usage is slightly lower than ITN coverage [5,20]. The household level ITN ownership, population level ITN coverage, actual ITN usage, and age-specific ITN usage in the study sites have been published previously, therefore will not be repeated in this paper [5,20].

#### Vector population density

Indoor resting mosquitoes were collected from an average of 60 and 100 houses in each intervention/control cluster in 2010 and 2011, respectively, by pyrethrum spray collection (PSC). Houses were selected randomly to ensure maximal spatial coverage. The number of female *Anopheles* mosquitoes was recorded for all intervention and control clusters. The indoor resting density was determined as the average number of anopheline females collected per house per night (f/h/n). The timings of the mosquito surveys were selected to represent the vector population during the different seasons both before IRS (February, 2010 and 2011) and after IRS (May and July, 2010 and 2011). Sample sizes of each site at each survey period are shown in Table 1.

Anophelines were morphologically identified using morphological keys and classified as *Anopheles gambiae* and *Anopheles funestus*[19]. Since vector density was calculated using pooled data of all species, no further classification work was conducted [7,19].

#### Asymptomatic parasite screening (APS)

A cross-sectional survey was conducted at each study cluster during February and March of 2010 and 2011, before the IRS and Bti application, to obtain the baseline malaria parasite prevalence data. The same survey was repeated in May 2010 and May and July of 2011 to evaluate the impact of the intervention. Finger-prick blood samples were taken from approximately 150 (in 2010) and 250 (in 2011) randomly selected individuals of different ages within each cluster for the detection of the *Plasmodium* parasite (Table 1). A thin and a thick smear were prepared for parasite detection and species identification. Blood smears were read by a Kenya Medical Research Institute (KEMRI) laboratory technician. Quality was controlled by additional readings of randomly selected slides [5]. Individuals found to be positive for malaria infection and with malaria symptoms were referred to local health facilities for free anti-malarial treatment according to Kenya government guidelines. Information on bed net usage for the previous night before the APS survey, for each participant, was recorded.

Parasite prevalence was calculated as the percentage of individuals in the surveyed population having positive slide readings during the survey. Prevalence was calculated for each survey occasion. Parasite was counted against 200 white blood cells and parasite density was calculated as number of parasites per μL assuming 8,000 white blood cells per μL. Gametocyte density was calculated the same way.

#### Active case surveillance (ACS) of malaria incidence

A cohort malaria incidence study was started at each cluster one month before IRS and Bti application. The cohort population under surveillance was on average 350 individuals in each cluster in 2010 and 450 individuals in each cluster in 2011 (Table 1). Home visits of the cohort were undertaken bi-weekly beginning in February and ended in the end of July and first week of August in 2010 and 2011, respectively. A clinical malaria case is defined as an individual with malaria-related symptoms (fever, i e, axillary temperature ≥37.5°C, chills, severe malaise, headache or vomiting) at the time of examination or one to two days prior to the examination with *Plasmodium* positive blood smear. In all study sites during each visit, individuals with either subjective (i.e. reported) or objective fever (axillary temperature >37.5°C) had blood smears prepared for the testing of malaria parasites. For participants with signs of malaria other than fever, a blood sample was taken to prepare a thin and a thick smear for parasite species identification. Blood smear tests were performed by the same KEMRI laboratory technician who read the APS slides. Clinical cases were referred to the local hospital or clinic for free treatment according to Kenya government guidelines. Individuals with missing information or lack of consistent information on bed net usage have been excluded from final ACS data analysis. Active case rate (ACR) was defined as the number of malaria cases per 1,000 population (denoted by ‰) per survey.

The co-ordinates of each household that participated in the PSC surveys and individual or household that participated in the APS and ACS surveys were taken using eTrex Venture hand set Geographic Positioning System (GPS) unit (Garmin International, Olathe, Kansas, USA). Participating households and individuals were divided into clusters according to prior intervention design and intervention/control assignments.

### Statistical analysis

For data analysis purposes, clusters were classified as intervention and control clusters; each cluster was further divided into two areas, targeted and non-targeted areas (see Figure 1). The targeted area within the intervention cluster was the targeted IRS area, and the rest of the intervention cluster was the non-targeted area (Figure 1). The targeted area within the non-intervention cluster was the area corresponding to the targeted IRS area in the intervention cluster, and the rest of the non-intervention cluster was the non-targeted area (Figure 1). The buffer zones are areas where samples were excluded from final data analysis so as to avoid edge effect (Figure 1). Based on the dispersion of the data, we chose to conduct our data analyses using pooled data of each group defined by study site, season, specific cluster, intervention/nonintervention zone, and with/without ITN, for example, parasite prevalence of population at Iguhu - prior to intervention 2010 - intervention cluster 1 - nonintervention zone - without ITN.

Changes in seasonal mean vector densities, parasite prevalence, and incidence rate of malaria cases detected via ACS before and after intervention were tested by the use of the functional additive model (FAM) [21]. FAM is a random effect additive model which allows for interactions among factors, using study sites (tests variation among sites), survey periods (before and after intervention), clusters (intervention and non-intervention), zones (intervention and non-intervention zones within a cluster to test targeted intervention effect), and bed net (using and not using), as well as the interactions among them as independent variables and using the outcomes from the corresponding non-intervention area as contrast for the dependent variable. Models were estimated using a co-ordinate descent Gause-Seidel backfitting procedure via general linear model (GLM) analysis. Statistical software JMP 9.0 (SAS Corporate, Cary, NC, USA) was used for data analysis.

### Scientific and ethical statement

Scientific and ethical clearance was given by the institutional scientific and ethical review boards of the Kenya Medical Research Institute, Kenya and the University of California, Irvine, USA. Written informed consent/assent (for minors under age of 18) for study participation was obtained from all consenting heads of households and each individual who was willing to participate in the study. Inclusion criteria were provision of informed consent/assent and no reported chronic or acute illness except malaria. Exclusion criteria were those who were unwilling to participate or infants under age of six months.

## Results

The descriptive statistics are summarized in Table 1. ICON™ spraying was conducted in 3,086 and 2,846 targeted houses in 2010 and 2011, respectively. Indoor resting vector density monitoring was conducted in 3,271 and 3,917 houses in 2010 and 2011, respectively. Blood samples were collected from 19,935 (5,790 in 2010 and 14,145 in 2011) individuals for asymptomatic parasite screening; parasite was detected from 522 and 1,418 slides in 2010 and 2011, respectively. ACS recruited 6,248 and 5,572 individuals in 2010 and 2011, respectively, with totals of 88,507 and 61,314 surveillance visits in 2010 and 2011, respectively; the numbers of malaria cases detected were 265 and 254 episodes in 2010 and 2011, respectively.

**Figure 2:**
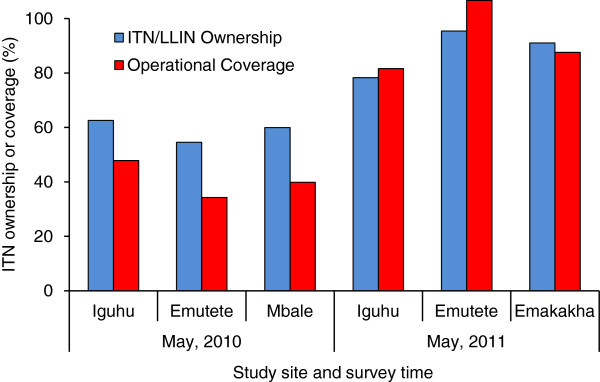
Insecticide-treated bed net ownership and population coverage in May, 2010 and May, 2011 in different study sites.

The ITN ownership survey was carried out in 2,487 households that covered a population of 9,389 individuals across the three study sites (Table 1). The household ITN ownership was on average 59.0% (ranging from 54.5-62.6%) in May, 2010 and it increased dramatically to 90.6% (ranging from 78.3-97.2%) in June, 2011 after the 2011 mass ITN distribution campaign (Figure 2). Population ITN coverage increased from 40.7% (range from 34.3-47.8%) in 2010 to 93.0% (range from 81.6–100%) in 2011 (Figure 2).

### Vector density

**Figure 3:**
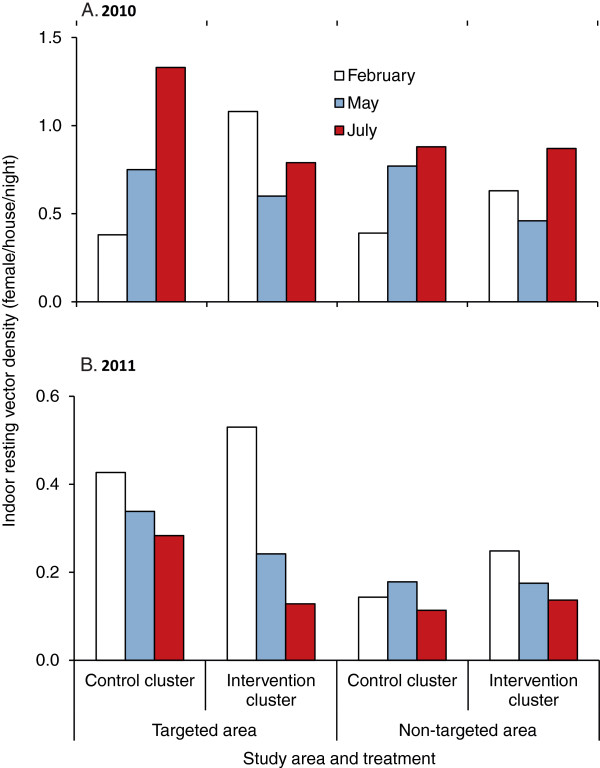
**Average *Anopheles *densities in different areas and different months in 2010 (A) and 2011 (B).**

The results of the 2010 intervention showed a mixed impact after the implementation of IRS. In the control clusters, anopheline densities showed an overall increase from February to May and further increase in July; vector densities in the intervention clusters decreased from February to May but had rebounded by July (Figure 3A). IRS alone (F _1,131_ = 3.45, P = 0.065) or ITN alone (F _1,131_ = 2.97, P = 0.087) showed a marginally significant impact on reducing vector densities.

When the targeted areas were further divided into ITN and non-ITN covered households in addition to IRS and non-IRS households, households were classified into four types: non-ITN and non-IRS; ITN but non-IRS; IRS but non-ITN; and, both IRS and ITN. Compared to non-IRS and non-ITN houses, there was a 25–38% reduction in relative risk in ITN-only houses, 65–68% reduction IRS-only houses, and 71-79% reduction in combined ITN and IRS houses. This trend in vector density change was similar for all study sites, although with variations of different magnitude (Additional file 1), and relative risk to vector density varied with different intervention methods (Additional file 2).

When vector densities were analysed, the results of the 2011 intervention showed a significantly reduced effectiveness compared to 2010 despite the added larval intervention using Bti in 2011. Vector densities showed decreasing trends at all clusters from February to May and further decreased in July, but the decrease in vector densities in the intervention clusters was more pronounced (Figure 3B). Both IRS (F _1,103_ = 0.37, P = 0.55) and ITN (F _1,103_ = 1.53, P = 0.22) did not show significant impact on vector densities. Inter-site variation in vector density has been observed (Additional file 1), and relative risk to vector densities varied with different season and patterns in relative risks were similar to that in 2010 (Additional file 2).

Despite the evident relative reductions in vector densities, backfitting selection of GLM analysis revealed that study site (2010 and 2011) and study season (before and after intervention) (2011) were the only significant factors that affected vector densities, and ITN and IRS were marginally significant in 2010 but not 2011 (Additional file 3). Backfitting selection also revealed that IRS (ICON), especially IRS in Iguhu compare to Emutete, had significant impact on reducing vector densities in 2010 but not in 2011; whereas ITN had significant impact on reducing post-intervention vector density in 2011 compare to prior-intervention. In other words, ICON spray in 2010 had significant added impact, but ICON + Bti intervention in 2011 only had modest impact on reducing vector population, i.e., ICON + Bti did bring vector densities down to prior intervention level during the traditionally peak transmission season (post-intervention season), but did not further reduce the absolute vector densities as has been achieved by previous intervention [7].

### Asymptomatic parasite prevalence

**Figure 4:**
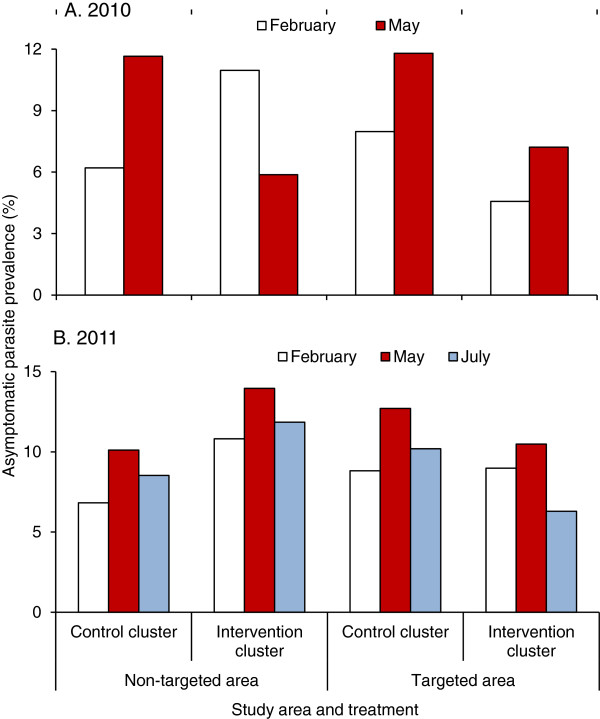
Average parasite prevalence in different areas and different months in 2010 (A) and 2011 (B).

Similar to the vector densities, parasite prevalence in the control clusters in 2010 increased significantly from February to May (Figure 4A). Parasite prevalence in the targeted areas of the intervention clusters decreased from an average of 11.0% in February 2010 to 5.9% in May 2010 (Figure 4A). Whereas parasite prevalence in the non-targeted area of the intervention clusters increased from an average of 4.6% in February 2010 to 7.2% in May 2010 (Figure 4A).

The trend in parasite prevalence was consistent for all study sites (Additional file 4), and relative risk to parasite infections varied with intervention methods (Additional file 5). Compared to the numbers for the no-ITN and no-IRS group, the ITN-only group showed a 40.0% reduction in relative risk in parasite prevalence from February to May 2010, the IRS-only group showed a reduction of 58%, and the group with combined IRS and ITN intervention yielded a 79% reduction in relative risk (Additional file 5).

The reduction in parasite prevalence was even lower after the 2011 intervention when compared to the numbers in the 2010 intervention. Compared with February 2011, parasite prevalence had increased in all clusters in May 2011, regardless of intervention or non-intervention status (Figure 4B). This trend in parasite prevalence was similar for all study sites, although with variations of different magnitude (Additional file 4), and relative risk to parasite infection varied with different season (Additional file 5).

In addition to the difference in parasite prevalence among study sites, backfitting selection of GLM analysis demonstrated that ITN and the interaction between ICON spray and study periods in 2010 had significant influence on parasite prevalence, which indicated that ICON spray reduced parasite prevalence significantly in 2010 (Additional file 6). The impact of ITN on reducing parasitaemia, analysed by GLM, was marginally significant and site-specific, while both ITN and IRS had no significant impact on gametocytaemia in 2010 (Additional file 6). Using the same method of analysis, the results of 2011 data illustrated that parasite prevalence showed a significant difference among study sites and ICON spray significantly reduced parasite prevalence, but the impact of ITN on reducing parasite prevalence was site-specific (Additional file 6). Stepwise analysis showed that differences in parasite densities in 2011 were marginally significant among different sites and among different seasons, but were significant when interactions among season, site, ITN, and ICON were considered (Additional file 6). ICON application in 2011 was significantly correlated with reduced gametocyte density (Additional file 6).

### Clinical malaria incidence

**Figure 5:**
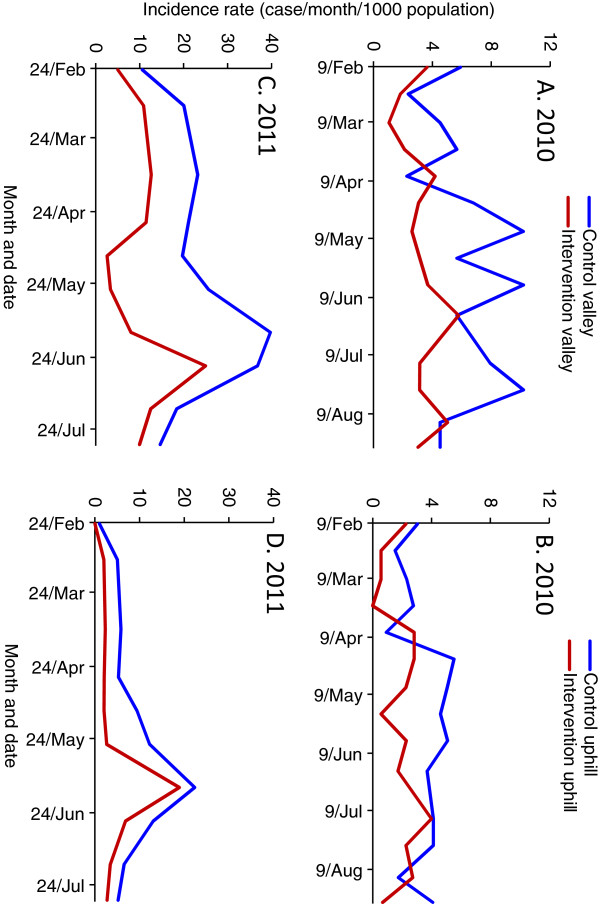
Incidence rate (cases/1000 population/survey) observed via active case surveillance in different areas before and after intervention in 2010 (A and B) and 2011 (C and D).

Due to the limited number of clinical cases detected, analysis of clinical malaria cases was based on clusters, and clusters were not further divided into intervention and non-intervention areas. In both 2010 and 2011, ACR increased in the non-intervention clusters before the intervention compared to after the intervention, which was a reflection of normal seasonal transmission fluctuations, whereas ACR decreased in the intervention clusters during the same periods (Figure 5). The average ACR in the non-intervention clusters in 2010 was 6.0‰ (per thousand people) per survey before intervention and it was 7.3‰ per survey after intervention. In contrast, the average ACR in the intervention clusters was 7.8‰ per survey before the intervention and 4.4‰ per survey after the intervention. Similarly, the average ACR in the non-intervention clusters in 2011 was 10.4‰ per survey before the intervention and 15.4‰ per survey after the intervention (Figure 5A). In comparison, the average ACR for the intervention clusters was 17.5‰ per survey before the intervention and 12.0‰ per survey after the intervention (Figure 5B). The overall increase (not changes before and after intervention) in ACR in 2011 was due to the change of study site (Additional file 7).

There were significant variations in ACR and their relative reductions among study sites, but changes in trends of ACR were similar for all study sites (Additional file 7). Mbale had the lowest ACR among the three sites in 2010 and Emakakha, the replacement of Mbale, had the highest ACR among the three sites in 2011, and ACR in both Iguhu and Emutete remained almost unchanged (Additional file 5).

Backfitting selection of GLM analysis on 2010 clinical case data indicated that site, season, and the interaction among study site, season (before/after intervention), and intervention measure (with/without ICON) had significant effect on clinical malaria incidence rate detected through ACS, but the effect of intervention itself was not statistically significant (Additional file 8). On the other hand, effects of study site, season, ITN, and the interactions among study site, season, and ITN were the significant factors affecting clinical case rate in 2011; the effects of ICON on clinical case rate were not statistically significant (Additional file 8).

## Discussion

Integrated management is considered to be the best strategy for malaria control, elimination, and eventual eradication, but precautions must be taken for the integration of different intervention measures [4,22-26]. Using additional intervention methods does not necessarily effectively and significantly magnify the impact. When ITN coverage was low, adding IRS as a complementary intervention measure had significant added impact on malaria transmission [7,27,28]. Compared with the conditions several years ago, ITN coverage in the study sites is considerably higher now, and indoor resting vector densities are relatively low [5]. Compared with similar IRS application trials in 2005, the effectiveness of the current IRS in both 2010 and 2011 was significantly reduced [7]. The 2005 IRS nearly eliminated the major malaria vectors, *An*. *gambiae* and *An*. *funestus*, in the IRS covered area, and IRS was effective for at least six months, with a 64% relative reduction in asymptomatic parasite prevalence. The reductions in vector density and parasite prevalence of the 2010/2011 IRS and Bti intervention were modest compare to the 2005 intervention, i.e., reduced efficacy. These results raise concerns about the possible causes of the reduced effectiveness of the IRS intervention: a) mediation of malaria transmission resilience to IRS by high coverage of ITNs; b) possible vector resistance to insecticide; and c) vector behavioural changes.

### ITN coverage

High ITN coverage clearly plays an important role in combating malaria transmission. It was believed that the use of ITNs may produce community-wide effects on the vector population and parasite prevalence even if ITN coverage was as low as 20% [17]. Modelling work showed that an 80% or higher ITN coverage may totally prevent malaria transmission [15]. Thus, at high ITN coverage, integrating IRS and Bti into the intervention matrix may have limited additional impact on malaria transmission. The ITN coverage in the study areas was less than 20% in 2004, and the impact of IRS on reducing malaria transmission was very high in 2005 [5,7]. The ITN coverage in the study sites were all above 80% in 2011, and the additional impact of applying IRS and Bti was significantly lower than that in 2005 and 2010. Therefore, it is possible that the impact of IRS and Bti on malaria transmission was mediated by ITN. However, other factors should not be ruled out definitively as long-term dynamic data showed a rebounding vector population in some of the study sites [5].

### Vector density monitoring

Previous studies show that the prolonged use of ITNs may force vectors to change their host feeding preference: a shift to exophagy (outdoor biting) and exophily (outdoor resting) for the two important malaria vectors in Africa, *An. gambiae* s.l. and *An. funestus*, and a shift in biting behaviour to early in the evening before people sleep under nets or biting and resting outdoors to avoid ITNs [3,29-33]. N’Guessan *et al* found that fewer *An. gambiae* entered the ITN- and IRS-treated experimental huts than the respective control huts, i e, ITN and IRS might act as repellent [33]. Such changes can drastically reduce the level of personal protection conferred by ITNs. These behavioural changes may have resulted from the selection of genetically inherited traits or, more directly, from plastic phenotypic adaptations in response to the increased coverage of ITNs and IRS. With wide coverage of expanded intervention methods such as described here, malaria transmission should suffer a precipitous decline mediated through profound effects on vector populations. However, due to vector behavioural changes, ITN and IRS alone or combined may not drive transmission rates further downward and lead to the goal of elimination. The results from this study show a significantly reduced impact even with combined intervention of ITN, IRS and Bti, which is rather different than predicted by mathematical models and by previous field observations [15,29,34]. It is not clear whether Anophelines in these places have changed their biting behaviour or not; further investigation is needed to monitor outdoor biting intensity and the possibilities of early evening biting. There is also an urgent need for alternative control methods to eliminate outdoor transmission should vector biting and resting outdoors.

### Larval Bti control

Previous studies showed that larval control using Bti may be promising in reducing larval population densities and malaria transmission [13]. However, use of regular formulation of Bti to control the larval population may be limited by its high operational costs and more importantly, by its short-lived effectiveness [35]. The effects of the current Bti formula last only about one week, and weekly re-application of Bti is impractical on large scales [13]. In this study, Bti had been applied two times before the major rainy season. Field monitoring showed that larval populations were nearly eliminated in most breeding habitats within a window period of about 10 days after Bti application, but the population rebounded to its original level after about two weeks of Bti application (Beilhe, unpublished data). There is a desperate need for a new formula of Bti that lasts for a whole transmission season of three to four months and will not be washed away by heavy rain, to reduce the financial cost as well as labour required, and to make it appropriate for large-scale application.

### Insecticide resistance management

Insecticide resistance can possibly be one of the key causes of the low effectiveness of IRS. Due to the same selection pressure, mosquitoes may develop resistance to insecticide after prolonged use of ITNs and IRS. Indeed, studies have found that vectors have already developed different levels of resistance to insecticides in various countries in southern and western Africa after only a few years of ITN usage [33,36-40]. More recently, resistance has been detected in *An*. *gambiae* s.s., *An*. *funestus*, and *Anopheles arabiensis* in the lakeshore area of western Kenya [41]. The key measurement of loss of efficacy associated with pyrethroid resistance is the loss of killing power, i.e., reduced mortality in malaria vectors of resistance populations.

Although insecticide resistance was not tested in this study (experiments underway), there was strong evidence to indicate possible insecticide resistance in both *An*. *gambiae* and *An*. *funestus* in the study sites [5]. The results of the 2005 IRS trail intervention in Iguhu indicated an elimination of *An*. *funestus* and near elimination of *An*. *gambiae* populations for up to six months within the IRS application area, at the time when *Anopheles*’ resistance to insecticide was not detected in the study areas [7,42]. In the current study, the reductions in indoor resting densities of *An*. *funestus* and *An*. *gambiae* in the IRS area were significantly reduced in both 2010 and 2011. Long-term population dynamics monitoring in the same area also showed a steady resurgence in indoor resting vector population since 2008 after an initial decline in population densities from 2004 to 2007, in spite of continuous increase in ITN coverage since 2004 [5]. The results indicate that mosquitoes are no longer sensitive to insecticide [5].

Prolonged use of bed nets has also been linked to the decline of *An*. *gambiae* s.s. relative to its sibling species *An*. *arabiensis*[29,34]. Studies in both coastal Kenya and the Lake Victoria shore areas of western Kenya demonstrated that long-term high coverage of ITN will eventually diminish the role of *An*. *gambiae* s.s. and *An*. *funestus* in malaria transmission [29,34]. Theoretically, such selective elimination diminishes the importance of *An. gambiae* s.s as the main malaria vector by intervention pressure and leaves those that are less vulnerable and residual to transmit malaria parasites. However, results of long-term population dynamics monitoring and results from the current study do not concur [5]. More importantly, vector resistance to insecticides was never considered in these previous optimistic predictions.

The limitation of this study is the limited length of intervention period. With continuous two years of intervention, the cumulative effects on parasite prevalence reduction may not be revealed, the actual positive effects of IRS may appear after quite a long time of continuous implementation of the control measure. Therefore, intervention design needs to be further improved so that long-term cumulative effect can be achieved.

## Conclusion

Although integrated management of malaria is considered to be the best approach to eliminate and eventually eradicate malaria, further investigations are needed to determine the components requiring integration and integration procedures. It seems that adding early season larval control and IRS to high ITN coverage has limited added impact on malaria transmission. Alternative control methods are needed to eliminate outdoor transmission and long-lasting Bti formulation is needed to sustainably suppress larval population. Nevertheless, currently available integrated malaria management strategies have immense impact on malaria transmission; however, there are also many more unprecedented challenges that lie ahead as malaria intervention intensifies. The road to malaria elimination and eradication in sub-Saharan Africa appears to be long and arduous [43,44].

## Competing interests

The authors declare that they have no competing interests.

## Authors’ contributions

GY, GZ, AKG and YAA conceived and designed the experiments. YAA, HEA, GZ, AD, CLW and LBB performed the experiments. AD and MCL were responsible for data management and quality control. GZ, AD and MCL analysed the data. GZ, GY, AD and AKG wrote the paper. All authors read and approved the final manuscript.

## Supplementary Material

Additional file 1 Indoor resting vector densities in different months at different study sites in 2010 (A) and 2011 (B). Click here for file

Additional file 2 **Relative risk to malaria vector in the targeted areas with different intervention methods in different months at different study sites.** Vector densities in houses without ITN and IRS/Bti was used as control (RR = 1.0). Click here for file

Additional file 3 Results of analysis of variance and backfitting model of mosquito densities. Click here for file

Additional file 4 Asymptomatic parasite prevalence at different study sites in 2010 (A) and 2011 (B). Click here for file

Additional file 5 **Changes in relative risks of malaria prevalence with different intervention methods in 2010 (A) and 2011 (B).** Parasite prevalence in population without ITN and IRS/Bti was used as control (RR = 1.0). Click here for file

Additional file 6 Results of analysis of variance and backfitting model of parasite prevalence and parasite and gametocyte densities. Click here for file

Additional file 7 Incidence rates (cases/1,000 population/survey) observed via active case surveillance in different areas and different study sites in 2010 (A) and 2011 (B). Click here for file

Additional file 8 Effects of intervention on clinical malaria incidence rate. Click here for file
