# Supplementary material for: Modest additive effects of integrated vector control measures on malaria prevalence and transmission in western Kenya
Source: Malar J. 2013 Jul 19;12:256. doi: 10.1186/1475-2875-12-256 (PMC3722122; doi:10.1186/1475-2875-12-256)

Additional file 1 Indoor resting vector densities in different months at different study sites in 2010 (A) and 2011 (B)

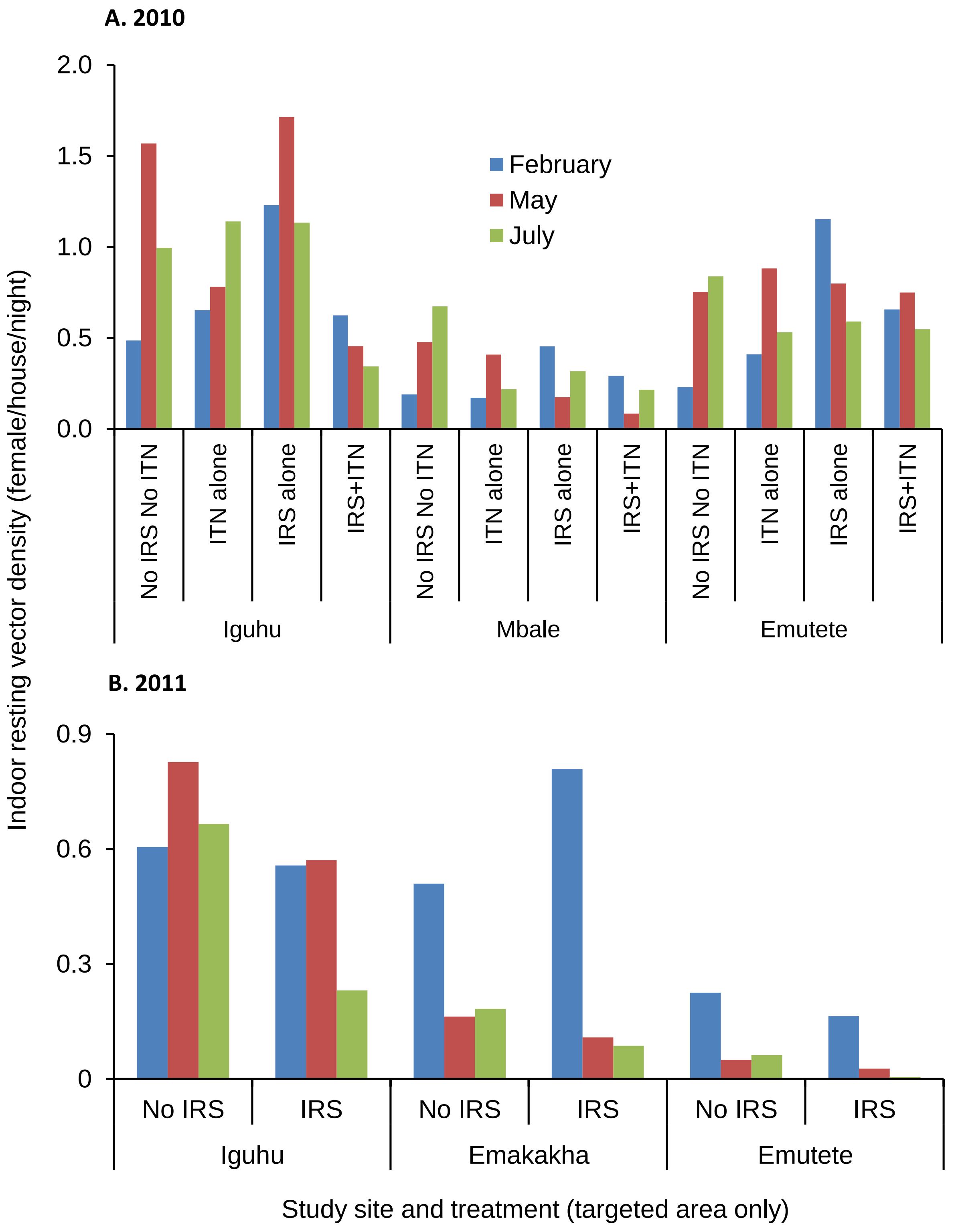

Supplement: Additional file 1 — Indoor resting vector densities in different months at different study sites in 2010 (A) and 2011 (B). [file 1475-2875-12-256-S1.pdf]
