# Supplementary material for: Modest additive effects of integrated vector control measures on malaria prevalence and transmission in western Kenya
Source: Malar J. 2013 Jul 19;12:256. doi: 10.1186/1475-2875-12-256 (PMC3722122; doi:10.1186/1475-2875-12-256)

Additional file 2 Relative risk to malaria vector in the targeted areas with different intervention methods in different months at different study sites. Vector densities in houses without ITN and IRS/Bti was used as control (RR = 1.0)

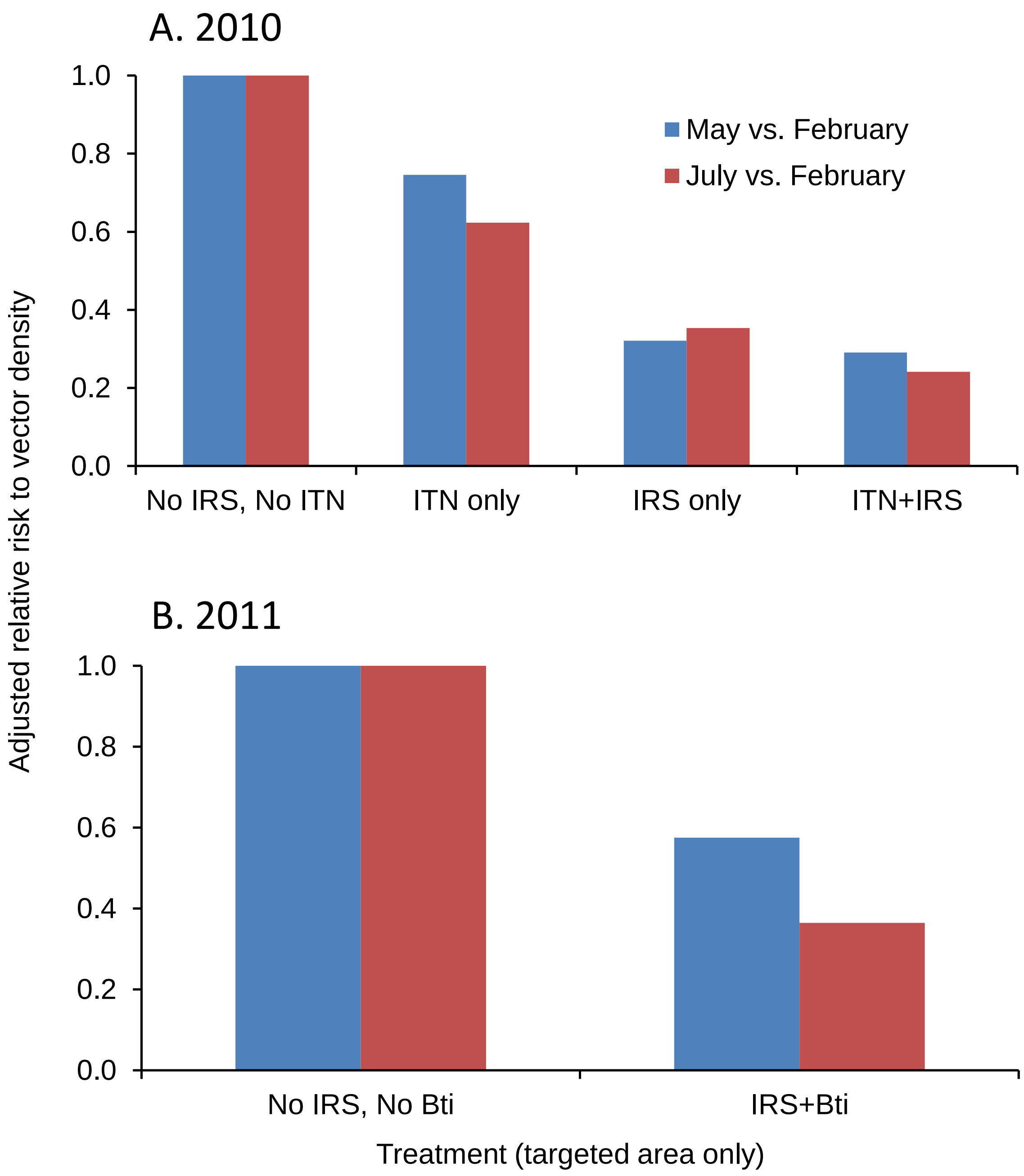

Supplement: Additional file 2 — Relative risk to malaria vector in the targeted areas with different intervention methods in different months at different study sites. Vector densities in houses without ITN and IRS/Bti was used as control (RR = 1.0). [file 1475-2875-12-256-S2.pdf]
