# Supplementary material for: Modest additive effects of integrated vector control measures on malaria prevalence and transmission in western Kenya
Source: Malar J. 2013 Jul 19;12:256. doi: 10.1186/1475-2875-12-256 (PMC3722122; doi:10.1186/1475-2875-12-256)
Supplement: Additional file 3 — Results of analysis of variance and backfitting model of mosquito densities. [file 1475-2875-12-256-S3.pdf]

Additional file 3: Results of analysis of variance and backfitting model of mosquito densities.

Table S3a. Categorical values encountered during processing.

Site (3 levels)	Emakhaha/Mbale , Emutete, Iguhu
Season (3 levels)	Prior intervention (February/March) , Post intervention (May), Late season (July/August)
ICON (2 levels)	Applied (yes), not applied (no)
ITN (2 levels)	With ITN (yes), without ITN (no)

2010 Data

Table S3b. Analysis of variance – full model. Dependent variable: anopheles adult density (female/house/night) in 2010. See Table S3a for independent variable descriptions.

Source	SS	df	MS	F	P
CONSTANT	100.832	1	100.832	63.772	0.000
SITE	26.828	2	13.414	8.484	0.000
SEASON	2.314	2	1.157	0.732	0.483
ICON	5.465	1	5.465	3.457	0.065
ITN	4.703	1	4.703	2.974	0.087
SITE*SEASON	2.336	4	0.584	0.369	0.830
SITE*ICON	19.101	2	9.551	6.040	0.003
SITE*ITN	1.627	2	0.814	0.515	0.599
SEASON*ICON	0.784	2	0.392	0.248	0.781
SEASON*ITN	1.855	2	0.928	0.587	0.558
ICON*ITN	0.225	1	0.225	0.143	0.706
SITE*SEASON*ICON	2.963	4	0.741	0.468	0.759
SITE*SEASON*ITN	1.437	4	0.359	0.227	0.923
SITE*ICON*ITN	1.167	2	0.583	0.369	0.692
SEASON*ICON*ITN	1.153	2	0.576	0.365	0.695
SITE*SEASON*ICON*ITN	6.012	4	1.503	0.951	0.437
Error	207.129	131	1.581		

Table S3c. Parameter estimates after backfitting selection at significance level of 0.05. Dependent variable: anopheles adult density (female/house/night) in 2010. See Table S3a for independent variable descriptions.

Term	Estimate	Std Error	t Ratio	Prob>|t|

Intercept	0.690	0.102	6.760	<.0001
Site (Mbale vs. Emutete & Iguhu)	0.371	0.102	3.650	0.000
Site (Emutete vs. Iguhu)	0.203	0.116	1.750	0.083
ICON (yes)	-0.243	0.095	2.560	0.011
Site (Iguhu vs. Emutete)*ICON (yes)	-0.406	0.115	3.520	0.001

2011 data

Table S3d. Analysis of variance – full model. Dependent variable: anopheles adult density (female/house/night) in 2011. See Table S3a for independent variable descriptions.

Source	SS	df	MS	F	P
CONSTANT	8.463	1	8.463	74.501	0.000
SITE	2.894	2	1.447	12.738	0.000
SEASON	1.721	2	0.860	7.573	0.001
ICON	0.042	1	0.042	0.366	0.546
ITN	0.174	1	0.174	1.533	0.218
SITE*SEASON	1.058	4	0.264	2.328	0.061
SITE*ICON	0.080	2	0.040	0.352	0.704
SITE*ITN	0.080	2	0.040	0.352	0.704
SEASON*ICON	0.177	2	0.088	0.779	0.462
SEASON*ITN	0.742	2	0.371	3.265	0.042
ICON*ITN	0.022	1	0.022	0.195	0.660
SITE*SEASON*ICON	0.168	4	0.042	0.369	0.830
SITE*SEASON*ITN	0.465	4	0.116	1.023	0.399
SITE*ICON*ITN	0.157	2	0.079	0.692	0.503
SEASON*ICON*ITN	0.012	2	0.006	0.051	0.950
SITE*SEASON*ICON*ITN	0.087	4	0.022	0.192	0.942
Error	11.701	103	0.114		

Table S3e. Parameter estimates after backfitting selection at significance level of 0.05. Dependent variable: anopheles adult density (female/house/night) in 2011. See Table S3a for independent variable descriptions.

Term	Estimate	Std Error	t Ratio	Prob>|t|
Intercept	0.339	0.030	11.230	<.0001
Site (Emutete & Emakhaha vs. Iguhu)	0.136	0.029	4.700	<.0001
Site (Emutete vs. Emakhaha)	0.080	0.034	2.370	0.019

Season (Post & Late vs. Prior)	0.111	0.029	3.860	0.000
ITN (yes)	-0.041	0.027	1.480	0.140
Site (Emutete vs. Emakhaha)*Season (Post & Late vs. Prior)	-0.082	0.035	2.310	0.023
Season (Post & Late vs. Prior)*ITN (yes)	-0.075	0.029	2.610	0.010
