# Supplementary figures and images for: Modest additive effects of integrated vector control measures on malaria prevalence and transmission in western Kenya
Source: Malar J. 2013 Jul 19;12:256. doi: 10.1186/1475-2875-12-256 (PMC3722122; doi:10.1186/1475-2875-12-256)

## Additional file 4 Asymptomatic parasite prevalence at different study sites in 2010 (A) and 2011 (B)

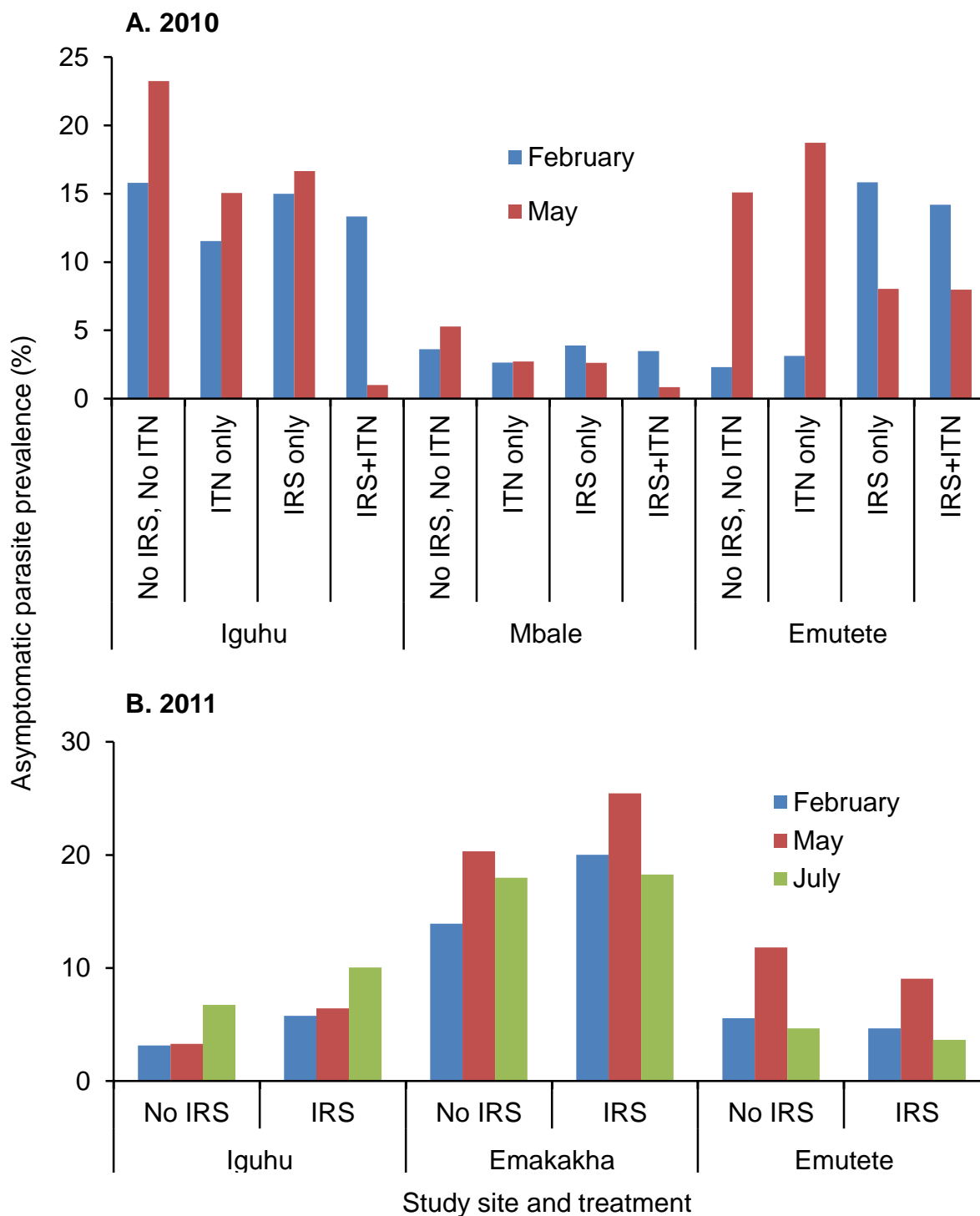

Supplement: Additional file 4 — Asymptomatic parasite prevalence at different study sites in 2010 (A) and 2011 (B). [file 1475-2875-12-256-S4.pdf]
