# Supplementary material for: Modest additive effects of integrated vector control measures on malaria prevalence and transmission in western Kenya
Source: Malar J. 2013 Jul 19;12:256. doi: 10.1186/1475-2875-12-256 (PMC3722122; doi:10.1186/1475-2875-12-256)

Additional file 5 Changes in relative risks of malaria prevalence with different intervention methods in 2010 (A) and 2011 (B). Parasite prevalence in population without ITN and IRS/Bti was used as control (RR = 1.0).

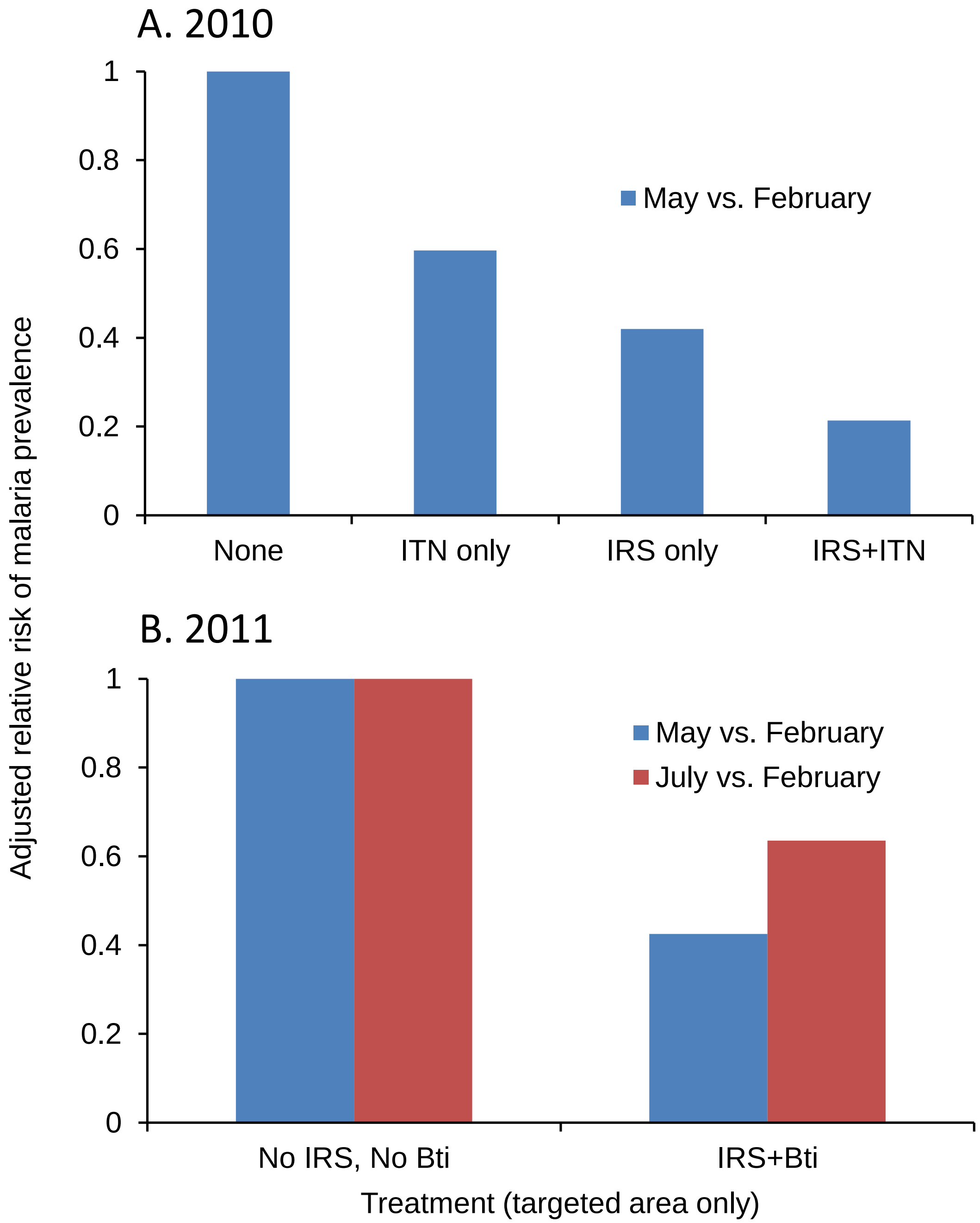

Supplement: Additional file 5 — Changes in relative risks of malaria prevalence with different intervention methods in 2010 (A) and 2011 (B). Parasite prevalence in population without ITN and IRS/Bti was used as control (RR = 1.0). [file 1475-2875-12-256-S5.pdf]
