# Supplementary material for: Modest additive effects of integrated vector control measures on malaria prevalence and transmission in western Kenya
Source: Malar J. 2013 Jul 19;12:256. doi: 10.1186/1475-2875-12-256 (PMC3722122; doi:10.1186/1475-2875-12-256)
Supplement: Additional file 6 — Results of analysis of variance and backfitting model of parasite prevalence and parasite and gametocyte densities. [file 1475-2875-12-256-S6.pdf]

Additional file 6: Results of analysis of variance and backfitting model of parasite prevalence and parasite and gametocyte densities.

2010 data

Table S6a. Analysis of variance – full model. Dependent variable: parasite prevalence in 2010. See Table S3a (Additional file 3) for coding of categorical variables.

Source	SS	df	MS	F	P
CONSTANT	4529.190	1	4529.190	132.281	0.000
SITE	1110.998	2	555.499	16.224	0.000
SEASON	14.692	1	14.692	0.429	0.516
ICON	121.887	1	121.887	3.560	0.065
ITN	16.920	1	16.920	0.494	0.485
SITE*SEASON	79.317	2	39.658	1.158	0.323
SITE*ICON	17.189	2	8.594	0.251	0.779
SITE*ITN	43.038	2	21.519	0.628	0.538
SEASON*ICON	40.514	1	40.514	1.183	0.282
SEASON*ITN	262.342	1	262.342	7.662	0.008
ICON*ITN	67.621	1	67.621	1.975	0.166
SITE*SEASON*ICON	17.589	2	8.795	0.257	0.775
SITE*SEASON*ITN	61.271	2	30.636	0.895	0.415
SITE*ICON*ITN	56.923	2	28.462	0.831	0.442
SEASON*ICON*ITN	53.045	1	53.045	1.549	0.219
SITE*SEASON*ICON*ITN	30.281	2	15.140	0.442	0.645
Error	1643.477	48	34.239		

Table S6b.Parameter estimates after backfitting selection at significance level of 0.05. Dependent variable: parasite prevalence (%) in 2010. See Table S3a (Additional file 3) for coding of categorical variables.

Term	Estimate	Std Error	t Ratio	Prob>|t|
Intercept	6.807	0.716	9.510	<.0001
Site (Mbale vs. Iguhu & Emutete}	-4.148	0.716	5.790	<.0001
Site (Iguhu vs. Emutete)	-1.924	0.837	2.300	0.025
Season (Post)	-0.306	0.675	0.450	0.652
ITN (yes)	-1.381	0.671	2.060	0.044
ICON (yes)	-0.383	0.674	0.570	0.572
Season (post)*ICON (yes)	-1.777	0.673	2.640	0.010

Table S6c. Parameter estimates after backfitting selection with mean parasitemia (parasites per microliter blood) as dependent variable ($F_{5,127} = 2.22$, $P = 0.056$). See Table S3a (Additional file 3) for coding of categorical variables.

Term	Estimate	Std Error	t Ratio	Prob>|t|
Intercept	233.401	73.143	3.190	0.002
Site (Mbale & Iguhu vs. Emutete)	88.368	73.143	1.210	0.229
Season (Prior)	7.907	66.556	0.120	0.906
ITN (yes)	-43.174	66.122	0.650	0.515
Site (Mbale & Iguhu vs. Emutete)*Season (Prior)	148.986	73.143	2.040	0.044
Site (Mbale & Iguhu vs. Emutete)*ITN(yes)	146.722	72.090	2.040	0.044

Table S6d. Parameter estimates after backfitting selection with mean gametocytemia (gametocyte per microliter blood) as dependent variable. See Table S3a (Additional file 3) for coding of categorical variables.

No variable has been selected at significant level of 0.05.

2011 Data

Table S6e. Analysis of variance – full model. Dependent variable: parasite prevalence (%) in 2011. See Table S3a (Additional file 3) for coding of categorical variables.

Source	SS	df	MS	F	P
CONSTANT	14605.775	1	14605.775	261.277	0.000
SITE	4765.532	2	2382.766	42.624	0.000
SEASON	85.283	2	42.641	0.763	0.469
ICON	538.150	1	538.150	9.627	0.002
ITN	74.092	1	74.092	1.325	0.252
SITE*SEASON	802.335	4	200.584	3.588	0.009
SITE*ICON	10.957	2	5.479	0.098	0.907
SITE*ITN	137.793	2	68.897	1.232	0.296
SEASON*ICON	202.328	2	101.164	1.810	0.169
SEASON*ITN	67.840	2	33.920	0.607	0.547
ICON*ITN	32.278	1	32.278	0.577	0.449
SITE*SEASON*ICON	471.341	4	117.835	2.108	0.085
SITE*SEASON*ITN	532.854	4	133.213	2.383	0.056
SITE*ICON*ITN	167.457	2	83.729	1.498	0.228
SEASON*ICON*ITN	354.767	2	177.383	3.173	0.046
SITE*SEASON*ICON*ITN	451.246	4	112.811	2.018	0.097
Error	5869.664	105	55.902		

Table S6f. Parameter estimates after backfitting selection at significance level of 0.05. Dependent variable: parasite prevalence (%) in 2011. See Table S3a (Additional file 3) for coding of categorical variables.

Term	Estimate	Std Error	t Ratio	Prob>|t|
Intercept	12.197	0.731	16.680	<.0001
Site (Iguhu & Emutete vs. Emakhaha)	6.072	0.731	8.300	<.0001
Site (Iguhu vs. Emutete)	1.491	0.826	1.810	0.073
ICON (yes)	-1.940	0.682	2.850	0.005

Table S6g. Parameter estimates after backfitting selection with mean parasitemia (parasites per microliter blood) as dependent variable ($F_{12,143}$ = 2.47, P = 0.006). See Table S3a (Additional file 3) for coding of categorical variables.

Term	Estimate	Std Error	t Ratio	Prob>|t|
Intercept	242.628	37.689	6.440	<.0001
Site (Emakhaha & Emutete vs. Iguhu)	61.393	33.974	1.810	0.073
Season (Prior & Late vs. Post)	65.846	36.999	1.780	0.077
ICON (yes)	-7.921	33.339	0.240	0.813
ITN (yeso)	-29.954	33.345	0.900	0.371
Site (Emakhaha & Emutete vs. Iguhu)* Season (Prior & Late vs. Post)	92.439	38.064	2.430	0.016
Site (Emakhaha & Emutete vs. Iguhu)*ICON (yes)	39.908	33.956	1.180	0.242
Site (Emakhaha & Emutete vs. Iguhu)*ITN(yes)	-31.231	33.959	0.920	0.359
Season (March & July vs. May)*ICON (yes)	24.798	36.923	0.670	0.503
Season (March & July vs. May)*ITN (yes)	-4.813	36.944	0.130	0.897
ICON (yes)*ITN (yes)	14.371	33.339	0.430	0.667
Season (Prior & Late vs. Post)*ICON (yes)*ITN (yes)	-98.220	36.923	2.660	0.009
Site (Emakhaha & Emutete vs. Iguhu)*ICON (yes)*ITN (yes)	-101.015	33.956	2.970	0.003

Table S6h. Parameter estimates after backfitting selection with mean gametocytemia (gametocyte per microliter blood) as dependent variable ($F_{1,154}$ = 4.80, P = 0.029). See Table S3a (Additional file 3) for coding of categorical variables.

Term	Estimate	Std Error	t Ratio	Prob>|t|
Intercept	2.171	0.460	4.71	<.0001
ICON (yes)	-1.009	0.460	2.19	0.029
