# Supplementary material for: Modest additive effects of integrated vector control measures on malaria prevalence and transmission in western Kenya
Source: Malar J. 2013 Jul 19;12:256. doi: 10.1186/1475-2875-12-256 (PMC3722122; doi:10.1186/1475-2875-12-256)
Supplement: Additional file 8 — Effects of intervention on clinical malaria incidence rate. [file 1475-2875-12-256-S8.pdf]

Additional file 8. Effects of intervention on clinical malaria incidence rate

Dependent variable: incidence rate (cases per 1,000 population per month). See Table S3a (Additional file 3) for coding of categorical variables, with Season replaced by prior- and post-intervention (post ICON) only.

2010 data

Table S8a. Analysis of variance – full model.

Source	SS	df	MS	F	P
CONSTANT	876.413	1	876.413	110.683	0.000
SITE	149.940	2	74.970	9.468	0.000
SEASON	87.900	1	87.900	11.101	0.001
ICON	7.356	1	7.356	0.929	0.338
ITN	19.032	1	19.032	2.404	0.126
SITE*SEASON	58.284	2	29.142	3.680	0.030
SITE*ICON	253.529	2	126.765	16.009	0.000
SITE*ITN	6.566	2	3.283	0.415	0.662
SEASON*ICON	13.022	1	13.022	1.645	0.204
SEASON*ITN	0.148	1	0.148	0.019	0.891
ICON*ITN	2.364	1	2.364	0.299	0.587
SITE*SEASON*ICON	1.594	2	0.797	0.101	0.904
SITE*SEASON*ITN	13.931	2	6.966	0.880	0.419
SITE*ICON*ITN	19.685	2	9.843	1.243	0.295
SEASON*ICON*ITN	17.692	1	17.692	2.234	0.139
SITE*SEASON*ICON*ITN	2.554	2	1.277	0.161	0.851
Error	554.277	70	7.918		

Table S8b. Parasite estimates after backfitting at significance level of 0.05.

Term	Estimate	Std Error	t Ratio	Prob>|t|
Intercept	2.638	0.298	8.840	<.0001
Site (Mbale vs. Emutete & Iguhu)	1.275	0.301	4.230	<.0001
Site (Emutete vs. Iguhu)	0.746	0.362	2.060	0.043
Season (Post)	-0.916	0.286	3.210	0.002
ICON (yes)	-0.331	0.292	1.130	0.260
Site (Mbale vs. Emutete & Iguhu)*Season (Post)	-0.902	0.296	3.050	0.003
Site (Emutete vs. Iguhu)*ICON (yes)	-2.035	0.363	5.610	<.0001

2011 data

Table S8c. Analysis of variance – full model.

Source	SS	df	MS	F	P
CONSTANT	20304.695	1	20304.695	157.800	0.000
SITE	15990.389	2	7995.194	62.136	0.000
SEASON	7206.567	1	7206.567	56.007	0.000
ICON	0.151	1	0.151	0.001	0.973
ITN	1965.002	1	1965.002	15.271	0.000
SITE*SEASON	11280.443	2	5640.222	43.834	0.000
SITE*ICON	14.262	2	7.131	0.055	0.946
SITE*ITN	7160.870	2	3580.435	27.826	0.000
SEASON*ICON	8.599	1	8.599	0.067	0.797
SEASON*ITN	9144.990	1	9144.990	71.071	0.000
ICON*ITN	1.773	1	1.773	0.014	0.907
SITE*SEASON*ICON	59.755	1	59.755	0.464	0.498
SITE*SEASON*ITN	10656.964	2	5328.482	41.411	0.000
SITE*ICON*ITN	76.712	1	76.712	0.596	0.443
SEASON*ICON*ITN	59.057	1	59.057	0.459	0.501
SITE*SEASON*ICON*ITN	91.549	2	45.775	0.356	0.702
Error	6948.367	54	128.673		

Table S8d. Parameter estimates after backfitting selection at significance level of 0.05.

Term	Estimate	Std Error	t Ratio	Prob>|t|
Intercept	34.450	2.171	15.870	<.0001
Site (Iguhu & Emutete vs. Emakakha)	27.149	2.171	12.500	<.0001
Site (Iguhu vs. Emutete)	0.704	1.451	0.490	0.629
Season (Post)	11.334	1.466	7.730	<.0001
ITN (yes)	4.685	1.472	3.180	0.002
Site (Iguhu & Emutete vs. Emakakha*Season (Post)	23.006	2.171	0.600	<.0001
Site (Iguhu vs. Emutete)*Season (Post)	-3.805	1.451	2.620	0.011
Site (Iguhu & Emutete vs. Emakakha)*ITN (No)	-17.994	2.171	8.290	<.0001
Season (Prior)*ITN (yes)	13.019	1.472	8.850	<.0001
Site (Iguhu & Emutete vs. Emakakha)*Season (Prior)*ITN (yes)	-22.658	2.171	10.430	<.0001
